# IL-15 Upregulates Telomerase Expression and Potently Increases Proliferative Capacity of NK, NKT-Like, and CD8 T Cells

**DOI:** 10.3389/fimmu.2020.594620

**Published:** 2021-01-18

**Authors:** Fiona Watkinson, Sandeep Krishan Nayar, Aradhana Rani, Christina A. Sakellariou, Oussama Elhage, Efthymia Papaevangelou, Prokar Dasgupta, Christine Galustian

**Affiliations:** ^1^ Peter Gorer Department of Immunobiology, School of Immunology and Microbial Sciences, Faculty of Life Sciences & Medicine, King’s College London, Guy’s Hospital, London, United Kingdom; ^2^ Urology Centre, Guy’s Hospital, London, United Kingdom

**Keywords:** interleukin-15, interleukin-2, telomerase, cell-signaling, adoptive cell therapy

## Abstract

Interleukin-15 (IL-15) is a cytokine that has been shown to expand CD8 T cell and natural killer (NK) cell populations, and therefore has potential for potentiating adoptive immune cell therapy for cancer. Previously, IL-15 has been shown to induce proliferation of CD8 memory T cells through activation of telomerase. Here, we investigated whether telomerase is also activated during the IL-15 mediated proliferation of NK and NKT-like (CD56+CD3+) cells. We also examined the extent that each of the three signaling pathways known to be stimulated by IL-2/IL-15 (JAK-STAT, PI3K-AKT Ras-RAF/MAPK) were activated and involved in the telomerase expression in the three cell types NK, NKT, or CD8 T cells. To assess cell proliferation and doubling, peripheral blood mononuclear cells (PBMCs) or isolated NK, NKT-like or CD8 T cells were incubated with varying concentrations of IL-15 or IL-2 for 7 days. CD8 T, NK, and NKT cell expansion was determined by fluorophore-conjugated antibody staining and flow cytometry. Cell doubling was investigated using carboxyfluorescein-succinimidyl-ester (CFSE). Telomerase expression was investigated by staining cells with anti-telomerase reverse transcriptase (anti-TERT). Telomerase activity in CD56+ and CD8 T cells was also measured *via* Telomerase Repeat Amplification Protocol (TRAP). Analysis of cellular expansion, proliferation and TERT expression concluded that IL-15 increased cellular growth of NK, NKT, and CD8 T cells more effectively than IL-2 using low or high doses. IL-15, increased TERT expression in NK and NKT cells by up to 2.5 fold, the same increase seen in CD8 T cells. IL-2 had effects on TERT expression only at high doses (100–1000 ng/ml). Proteome profiling identified that IL-15 activated selected signaling proteins in the three pathways (JAK-STAT, PI3K-AKT, Ras-MAPK) known to mediate IL-2/IL-15 signaling, more strongly than IL-2. Evaluation by signaling pathway inhibitors revealed that JAK/STAT and PI3K/AKT pathways are important in IL-15’s ability to upregulate TERT expression in NK and NKT cells, whereas all three pathways were involved in CD8 T cell TERT expression. In conclusion, this study shows that IL-15 potently stimulates TERT upregulation in NK and NKT cells in addition to CD8 T cells and is therefore a valuable tool for adoptive cell therapies.

## Introduction

Advances in the understanding of anti-cancer immunity has resulted in the successful use of immunotherapies against a number of cancers with checkpoint inhibitor antibodies such as the anti-CTLA-4 antibody Ipilumimab,(Yervoy™) and anti-PD1 antibodies Pembrolizumab, (Ketruda™) and Nivolumab, (Opdivo™) becoming FDA approved for a number of cancers (1). Alongside the development of these agents, adoptively transferred immune cell therapies, which have been trialed for over 20 years with disparate results, are now becoming more successful with the use of the chimeric antigen receptor (CAR) T cells, dendritic cells and allogeneic or syngeneic NK cells (2, 3). However, not enough is known about the lifespan of these cells, how long they remain active, and whether they can withstand the tumor microenvironment without being rendered anergic or regulatory.

IL-2 and IL-15 are cytokines that have been used to expand these immune cells before adoptive transfer, or for use as adjuvants to these cells once administered. Both cytokines can stimulate the proliferation of NK cells. IL-2 has been FDA approved for single agent administration to patients with renal cell carcinoma and melanoma (4), while, IL-15 has been administered in a number of first in man studies to patients with metastatic cancer (5, 6). However, IL-15 is believed to be favorable for use in patients compared with IL-2 due to its relative lack of toxicity, such as the lack of major resulting vascular leak syndrome (7). Additionally, unlike IL-2, IL-15 does not significantly affect the numbers and function of regulatory T (Treg) cells, which are stimulated by various cancers to induce suppression of CD8^+^ T cells and NK cells (8, 9). IL-15 can also increase the numbers and survival of both naïve and memory CD8 T cells (10). IL-15 also stimulates proliferation of NK and NKT cells (11–14), and IL-15 knockout mice have been shown to have decreased total numbers of these cell types (15). Furthermore, IL-15 was recently selected from a large panel of cytokines as the only agent that can significantly expand NK and CD8 T effector populations in the presence of prostate tumor cells (16).

The IL-15 receptor is composed of a β subunit (IL-2R/15Rβ/CD122) and a γ subunit (CD132) that is shared with the IL-2 receptor, and a distinct α subunit (IL-15Rα) giving the receptor specificity to the IL-15 cytokine (14). Three separate pathways are known to be activated by IL-15 ligation to its receptors: the JAK-STAT, the PI3K-AKT, and the Ras-MAPK pathways: Strong proliferative signals are induced *via* JAK/STAT and Ras/MAPK signaling pathways, and cell death is prevented by increasing anti-apoptotic proteins, such as Bcl-2 and Bcl-xL, and decreasing pro-apoptotic proteins such as Bim through activation of the PI3K pathway (17).

IL-15 is thought to act *via* a number of mechanisms to increase immune effector cell longevity. One such mechanism in CD8 T cells is through stimulation of telomerase. Telomerase is an enzyme that extends telomere length. Telomeres are DNA repeats found at the end of chromosomes that play a protective role in preventing genomic instability by blocking end-to-end fusion of chromosomes during cell division. These telomere repeats shorten after each cell replication cycle and eventually deplete leaving the chromosome ends to become exposed. Subsequently, genome instability occurs resulting in apoptosis (18). Telomerase activity has been shown to be important in the regulation of telomere length and consequently, replicative life span of lymphocytes in a controlled manner: Over-activation of the enzyme is a mechanism by which cancer cells can proliferate indefinitely. Telomerase consists of telomerase RNA (TERC) and telomerase reverse transcriptase (TERT) (19). Whereas TERC provides a template for telomere synthesis and is widely expressed in mammalian cells, TERT expression is restricted to cells with telomerase activity, including lymphocytes, and undergoes regulation *via* transcription, mRNA splicing and translation. Therefore TERT is the rate-limiting component of the reaction (20).

IL-15 has been shown to increase CD8^+^ memory T cell longevity by inducing telomerase activity through JAK3 and PI3K signaling pathways (21). The products of these pathways, NFκB, STAT3, c-Myc, and others have been identified as transcription activators that target the TERT promoter to restore and maintain telomere length for continuous cell proliferation and longevity (22).

In this study we examined whether IL-2 or IL-15 upregulate telomerase expression in NK and NKT-like cells in addition to CD8 T cells. We chose to study NKT-like cells, with the phenotype CD56+CD3+ and not other types of NKT cells (iNKT cells and type II NKT cells) due to their ability to be stimulated without antigenic restriction. Classical invariant NKT (iNKT) cells are CD1d-restricted V*α*24/V*β*11^+^ *α*-galactosylceramide (*α*-GalCer)-reactive T cells and Type II NKT cells are thought to have regulatory functions rather than an anti-tumor immune functionality whereas NKT-like cells are CD1d-independent and express diverse T-cell receptors (TCRs) (23). We also chose NKT-like cells due to reports of their strong toxicity against tumors (24), a role in treating hematopoietic malignancy (25) and the association of the progression of some cancers with a lack of this cell population (26).

There are, however, reports that CD8+NKT-like cells can inhibit the T-cell response by killing antigen bearing DCs (27). This also may explain the association of acute T−cell−mediated renal allograft rejection (ACR) with reduced infiltrating NKT-like cells. However, it has also been reported that they will kill antigen dependent myeloid suppressor cells (24). Their dual function may be therefore be beneficial in cancer patients with allografts.

Our investigation was additionally based on using bulk CD8 T cells, consisting of both naïve and memory cells, isolated from peripheral blood mononuclear cells, and not CD8 memory T cells due to the use of both memory and naïve T cells in adoptive therapies and the finding that naïve T cells may be as useful as memory T cells in these therapies because of their lower level of “exhaustion”, and greater ability to sustain replicative potential correlative to telomere length (28).

We investigated which of the three known pathways (JAK-STAT, PI3K-AKT Ras-MAPK) were activated in the each of the three cell types by IL-15 or IL-2 and the role of these pathways in the telomerase activity of these cells. We also compared the ability of IL-2 and IL-15 to expand and increase the proliferation and doubling capacity of NK, NKT, and CD8 T cells.

The study aimed to identify the optimal doses of IL-2 and IL-15 for use in expanding NK and NKT-Like, and CD8– T cells for adoptive transfer therapies and to define which pathways were most important for the effective proliferation of these cells.

## Materials and Methods

### Cytokines Used

Recombinant human IL-15 and IL-2 were obtained from PeproTech EC Ltd. (London, UK) and reconstituted from lyophilized protein to give a stock concentration of 10 μg/ml in sterile PBS.

### Isolation of Peripheral Blood Mononuclear Cells

Blood was collected from blood cones obtained from anonymized healthy donors (Blood Transfusion Service, NHS Blood and Transplantation), and PBMCs were isolated as previously described (29) and then prepared at 3 x 10^6^ cells/ml in culture medium containing Roswell Park Memorial Institute medium (RPMI) (Gibco, UK), fetal bovine serum (FBS) (Life Technologies, Paisley, UK), L-glutamine (Gibco, UK), mycoKill (PAA, UK), pen-strep (Gibco, UK) and gentamycin (Sigma, UK) (Complete medium). Cell solutions were transferred to 175 cm^2^ flasks and left to incubate at 37°C in a humidified 5% CO_2_ atmosphere for 3 h. The non-adherent PBMCs were then centrifuged at room temperature for 5 min, the supernatant was discarded and the pellets were re-suspended in complete medium for the proliferation assays.

### Preparation of Purified NK, NKT-Like, and CD8 T Cells

PBMCs prepared from the anonymized blood cone donors were used to isolate purified cell populations. CD56+ cells, NK cells, NKT-like cells, and CD8 T cells were isolated using kits 130-050-401, 130-092-657, 130-093-064, and 130-096-495 respectively from Miltenyi Biotech (Woking, UK) Cells were isolated according to the manufacturer’s instructions before treatment with our agents. Cells were checked for purity by staining with anti-CD56, anti-CD3, and CD8 fluorophore conjugated antibodies (clones MEM-188), (clone HIT1A) BioLegend UK, and clone BW135/80) (Miltenyi Biotech, Woking, UK) respectively followed by flow cytometric analysis using a BD FACSCalibur (BD Biosciences, Poole, Dorset, UK). Cells were prepared at a concentration of 8 x 10^5^ cells/ml for proliferation studies or cell signaling analysis.

### Lymphocyte Proliferation Assays

One ml of isolated non-adherent PBMCs or purified NK, NKT-Like or CD8 T cells (8 x 10^5^ cells) prepared in complete medium was placed in multiple wells of 24-well plates. 10 µl of differing concentrations of IL-15 or IL-2 diluted in phosphate buffered saline (PBS) (Severn Biotech Ltd, UK) were added to the wells or PBS was added as a negative control. Plates were then left to incubate at 37°C in a humidified 5% CO_2_ atmosphere for 7 days with additional doses of cytokines added at day 4.

### Surface Antibody Staining

Cells were removed from wells after the incubation period and transferred to Facs tubes for staining with antibodies for FACS analysis using standard protocols to detect surface markers (https://www.biolegend.com/protocols/cell-surface-flow-cytometry-staining-protocol/4283/). The antibodies used were fluorophore conjugated anti-CD3, anti-CD56, anti-CD4 (clones MEM-188, HIT1A, clone A161A1, respectively, BioLegend, UK), and anti-CD8 antibodies(clone BW135/80, Miltenyi, UK). Antibody staining was analyzed using a BD Calibur Flow cytometer (BD Biosciences Poole, Dorset, UK).

### Determination of Cell Expansion

Percentage expressions of NK (CD56^+^/CD3^-^) cells, NKT-Like (CD56^+^/CD3^+^) cells, CD4 and CD8^+^ T cells in the lymphocyte populations were analyzed through flow cytometry using CellQuest (BD Biosciences, USA) or FlowJo software (FlowJo, USA). Percentage gated values at quadrants corresponding to the different cell types were taken to compare cell expansion at the different IL-15 concentrations. The lymphocytes were gated on live cells within the lymphocyte population. Viability of the lymphocyte populations in the presence of the cytokines was measured using a live/dead stain (Live/Dead Fixable Near-IR Dead Cell staining (ThermoFisher Scientific, Paisley, UK) with analysis using the FACs canto flow cytometer BD Biosciences, Dorset UK).

### Determination of Cell Replicative Capacity *via* CFSE Staining

The Carboxyfluorescein succinimidyl ester (CFSE) Cell Proliferation Kit (PromoKine, Germany) was used to form a stock concentration of 5mM CFSE, which was diluted to 1mM using PBS. Following incubation of isolated PBMCs at 37°C for 3 h, the non-adherent cells were washed by centrifugation for 5 min at room temperature and the pellets were re-suspended in pre-warmed (37°C) CFSE solution in PBS. The cells were incubated for 15 min at 37°C to label the cells. The labeled cells were then centrifuged for 5 min at room temperature and the pellets were re-suspended in culture medium at a concentration of 8 x 10^5^ cells/ml ready for incubation with cytokines.

Surface antibody staining and flow cytometry was carried out following incubation with cytokines. Histograms of cell count against CFSE expression were set for each cell type at each concentration of cytokines. The geometric mean fluorescence was taken from each histogram to quantify the expression of CFSE and the number of cell divisions per population was calculated from the decrease of the Geometric mean where a 50% decrease was taken as 1 cycle of cell division.

### Determination of Telomerase Expression *via* TERT Staining

Following incubation with cytokines and subsequent surface antibody staining an intracellular staining protocol was carried out using a Cytofix/Cytoperm kit (BD Sciences USA) according to manufacturer’s instructions whereby APC-conjugated anti-TERT antibody (Bioss, USA) was incubated with the cells. Telomerase expression, as measured by TERT expression was measured for each cell type by analyzing the geometric mean values from histogram plots of cell count against TERT expression for each cell population at each concentration of IL-15 or IL-2. The LNCaP cell line purchased from ECACC (Public Health of England, Salisbury, UK), was used as a positive control for the expression of HTERT in selected experiments. Cells were cultured in RPMI 1640 medium (Sigma Aldrich, Poole, UK) supplemented with 2 mM glutamine, 1% antibiotic antimycotic solution and 10% fetal bovine serum (ThermoFisher Scientific, Paisley, UK). The cells were stained with anti-HTERT as above without previous treatment or surface antibody staining and were washed 3 times with PBS before antibody staining.

### Telomere Repeat Amplification Protocol

Purified CD56^+^ cells or CD8 T cells isolated by Miltenyi microbeads were prepared at 8 x 10^5^ cells/ml in 24 well plates with different concentrations of IL-15 for 7 days. The telomerase activity of the cells was assessed by a TRAP assay using the Telomerase PCR ELISA kit (Roche, Germany) according to the manufacturer’s protocol.

### Analyses of Cytokine Induced Phosphorylation of Proteins From the JAK/STAT/MEK and PI3K Pathways

Purified effector cells were prepared in RPMI complete medium at a concentration of 8 x 10^5^ cells per ml and incubated for 24 h in 6-well plates with either 0.1 ng/ml IL-2, 1000 ng/ml IL-2, 2.5ng/ml IL-15 (for optimal NK cell proliferation) or 50 ng/ml IL-15 (for optimal NKT-like or T cell proliferation). Cells were then lysed in Lysis buffer containing protease inhibitors and phenylmethanesulfonyl fluoride (PMSF) and lysates were prepared according to the manufacturers protocol for the Human Phospho-Kinase Array Kit (R&D Systems, Abingdon, Oxford, UK). Protein concentration of samples was quantified using a Coomassie (Bradford) Protein Assay Kit (ThermoFisher Scientific, Dartford, UK). Protein samples were stored at -80°C until protein analysis was continued.

Using the manufacturers protocol for the Human Phospho-Kinase Array (R&D Systems, Abingdon, Oxford, UK), cell lysate samples from each treatment were applied to a Part A membrane and a Part B membrane, containing complementary antibodies corresponding to different signaling proteins. The prepared membranes were analyzed using an ImageQuant LAS 4000 mini machine and the software ImageQuant 4000 (Ge Healthcare, Amersham UK). Image Studio Lite software analyzed the quantity of intensity of each membrane dot produced in these images that correlated to a specific protein. Each protein from each cell type was represented by two dots with their average being used to develop bar charts with error bars and a dot diagram to make a comparison between the low dose IL-2 control, IL-2, and IL-15. This procedure was repeated again with an alternative donor for all cell types to validate the results.

To confirm the results of the array, western blots were carried out with the proteins showing significant changes in phosphorylation with the different cytokines. Lysates were mixed with 4ul of 10x reducing agent (NuPAGE Sample Reducing Agent) and 10ul of 4x SDS-PAGE sample loading buffer (LDS 4x Sample buffer) (ThermoFisher scientific Dartford, UK) and subsequently heated at 95°C for 5 min and spun briefly. Samples were resolved on 4%–12% Bis-Tris gels and transferred to the iBlot nitrocellulose membrane using the iBlot Dry Blotting System (Invitrogen, UK). Western blots were performed using p38α (T180/Y182), CREB S133, ERK1/2 (T202/Y204, T185/Y187), AMPKα1 (T183), STAT5a (Y694), STAT5a/b (Y694/Y699), P53 (S392), AKT1/2/3 (S473), AKT1/2/3 (T308), Hsp60, mTOR (Ser2448), and GAPDH as the primary antibodies (Cell Signal, London, UK) and a HRP-linked anti-rabbit IgG antibody as the secondary antibody. Detection of the proteins were done using ECL chemiluminescence reagent (GE Healthcare, Amersham, UK). Where required, membranes were stripped using the western blot stripping buffer for 15 min and reprobed with another antibody. Blots were photographed and saved as TIF files from the ImageQuant LAS 4000 mini (GE healthcare, UK).

### Effects of Signaling Pathway Inhibitors on TERT Expression in the NK, NKT-Like, and CD8 T Cells

Non-adherent PBMCs were aliquoted at 8 x 10^5^ cells/ml in 24-well plates together with the optimal concentrations of cytokines found to increase TERT expression for each cell type (NK, NKT-like or CD8 T cells), or controls (either PBS alone or 0.1 ng/ml IL-2) for 7 days either alone or in the presence of inhibitors of each of the three signaling pathways known to be impacted by IL-2/IL-15. The CP690550 JAK1/3 inhibitor (BD Biosciences, Poole, Dorset, UK), was used at 10 µM for JAK1 inhibition and 0.1 µM for JAK3 inhibition (30). The LY-294002 hydrochloride PI3K/AKT inhibitor (Sigma, UK) (31) was used at 4 µM and the MEK/ERK inhibitor U0126 (32) (Promega, Southampton, UK) at 10 µM.

### Statistical Analyses

Data were plotted either as raw values of percentage expression or normalized to the untreated control of each experimental donor where there were large variances between donors to allow for more accurate comparison between experiments. Raw or normalized mean values along with their standard errors were calculated for each experiment. Ordinary one-way ANOVA was carried out using the program GraphPad Prism 8 (GraphPad Software, La Jolla, CA) for all data to determine a 95% level of statistical significance. Post hoc Newman Keuls multiple comparison tests were carried out where appropriate for comparison of data from various concentrations of cytokines against control data without cytokines. The results are all expressed as differences seen from lymphocytes taken from the same donor and treated with the different cytokines and doses, with analysis as repeated measures anovas. In the figure legends where n numbers are mentioned, these describe experiments from separate donors. Therefore, if n = 6, six separate experiments were performed from six independent donors.

## Results

### IL-15 Is Superior to IL-2 in Expanding NK, NKT-Like, and CD8 T Cells

**Figure 1:**
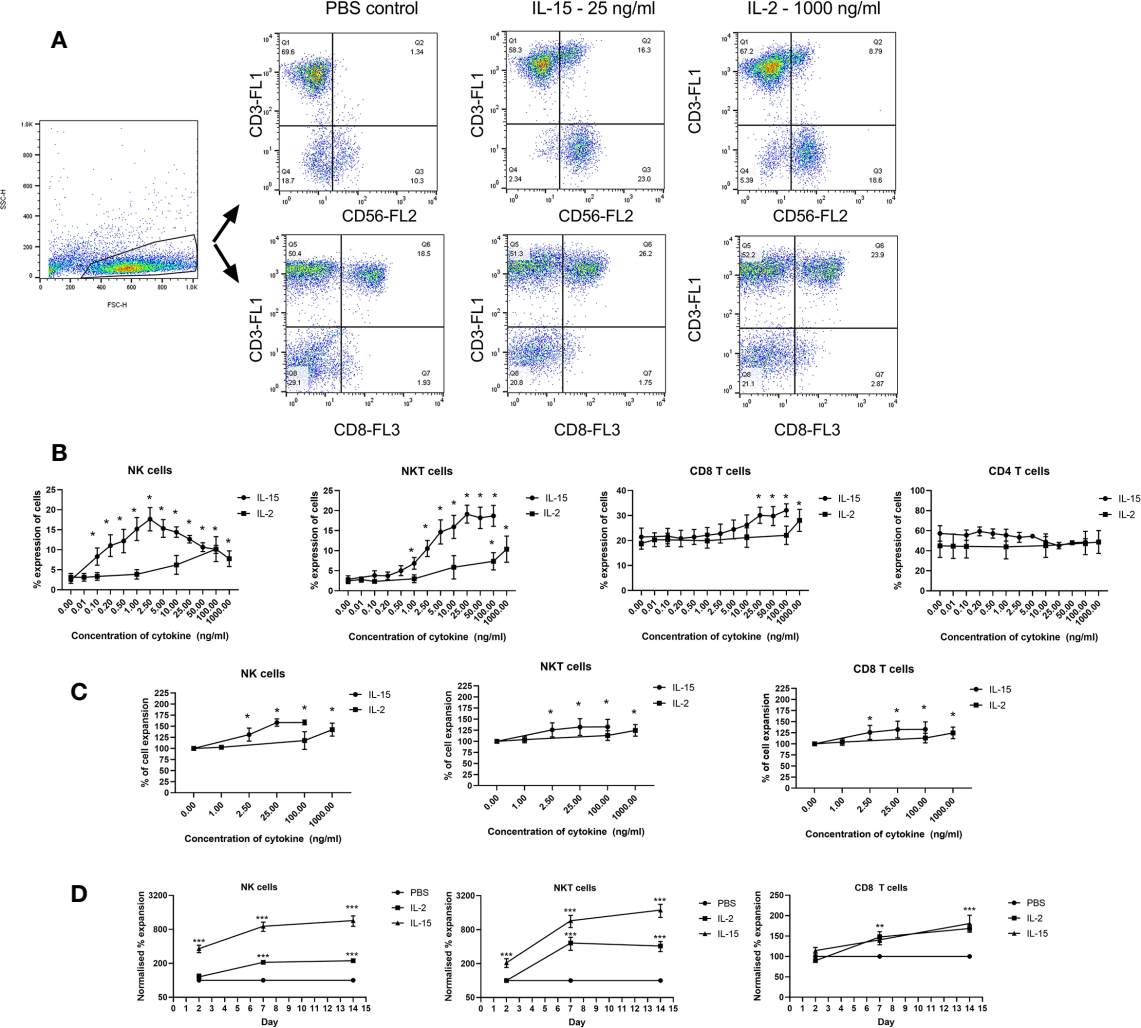
Expansion of immune effector cell populations treated for 7 days with IL-15 at concentrations from 0.01 ng/ml to 100 ng/ml or IL-2 from 0.1 to 1000 ng/ml with PBS as a control. **(A)** Gating strategy for NK cells, NKT-like cells and CD8 T cells in the PBMC population showing the significant increase in CD56+CD3- cells (NK cells), CD56+CD3+ (NKT cells) and (CD3+CD8+) (CD8 T) cells in non-adherent PBMCs treated with IL-15. **(B)** displays quantitated expression of NK, NKT, CD8 T cells and CD4 T cells in lymphocytes treated with different doses of IL-15 or IL-2. **(C)** shows increased total percentage of purified NK, NKT, and CD8 T cells upon incubation with IL-2 or IL-15. **(D)** shows the expansion of the three cell types over a time period with time points at days 2, 7, and 14 using IL-15 at 2.5 ng/ml for NK cells and 25 ng/ml with a more pronounced expansion with IL-15 compared to IL-2 in NK and NKT-like cells. Results are expressed as % of the expansion of the untreated lymphocytes and represent means ± SEM from six separate experiments (*p < 0.05, **p < 0.01 and ***p < 0.001 by one-way ANOVA and Newman Keuls post-hoc analysis).

IL-2 and IL-15 induced expansion of NK, NKT-like and CD8 T cells in populations of non-adherent PBMCs was investigated by determining the percentage expression of these cells in a population of gated lymphocytes after 7 days incubation with the cytokines. **Figure 1A** displays the flow cytometric gating to measure the NK, NKT-like and CD8 T cells within the non-adherent PBMC population. **Figure 1B** shows that IL-15 upregulated expression significantly from a concentration of 0.1 ng/ml in NK cells, 1 ng/ml in NKT-like cells and 25 ng/ml in CD8 T cells. A ten-fold maximal increase in expression occurred at 2.5 ng/ml in NK cells, and at 25–100 ng/ml in NKT-like cells. CD8 T cells were significantly increased by 1.5 fold from 25 ng/ml to 100 ng/ml.

IL-2 incubation resulted in a more modest increase in expansion with a 5-fold increase occurring from 100–1000 ng/ml in NK and NKT-like cells whereas CD8 T cells were expanded by 1.3 fold at 1000 ng/ml. CD4 T cells were not expanded at any dose either by IL-15 or IL-2. Purified NK, NKT, and CD8 T cells also show increased expansion when treated with IL-15 or IL-2 (**Figure 1C**). NK cells, NKT-like cells and CD8 T cells were isolated before we treated then with our agents as we wanted to examine the effects of the cytokines on the growth of the individual cell types. We also examined cell expansion at days 2, 7, and 14 to compare the growth rate obtained by the two cytokines (**Figure 1D**). These demonstrate that for NK and NKT-like cells, IL-15 is superior to IL-2 at increasing cell expansion and whereas IL-2 induced growth plateaus at day 7, NK, and NKT cell growth increases beyond 7 days although growth plateaus for IL-15 as well at 14 days.

**Figure 2:**
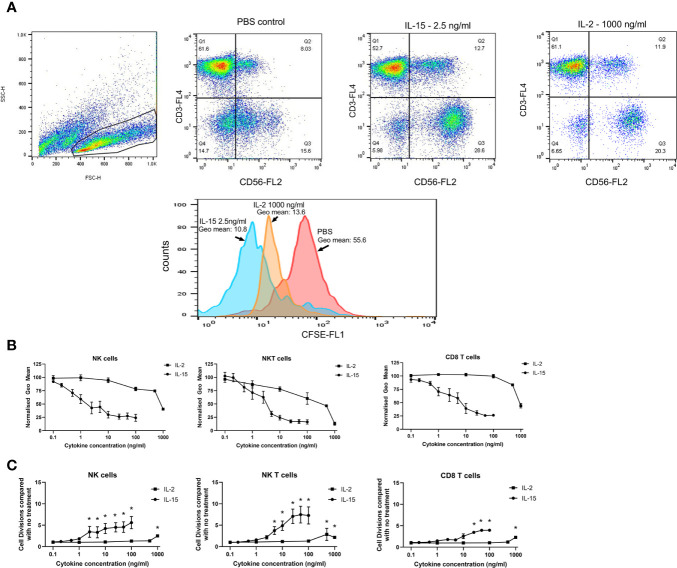
Effects of IL-15 and IL-2 on cell doubling capacity. Non-adherent PBMCs premixed with CFSE were treated for 7 days with concentrations of IL-15 from 0.1–100 ng/ml and IL-2 from 0.1 to 1000 ng/ml with PBS as a control. **(A)** displays the dot plots and a representative histogram showing the representative CFSE staining of a population of CD56+CD3- NK cells in the PBMCs. CFSE staining is measured as the geometric mean of the gated NK cell population and a decrease in the geometric mean signifies an increase in cell division. **(B)** shows the effects of IL-15 and IL-2 on NK, NKT-like and CD8 T cell proliferation as expressed by the normalized geometric mean fluorescence of CFSE after 7 days. **(C)** shows the number of cell divisions observed above that of the control cell population (with no cytokines). The numbers of cell divisions were calculated from the geometric mean fluorescence intensity of the cells by using 1 cell division as equivalent to a 50% decrease in the geometric mean of CFSE. Results represent means ± SEM from six separate experiments (* p < 0.05 by one-way ANOVA and Newman Keuls post-hoc analysis).

The gated lymphocytes analyzed represent the live lymphocyte population as confirmed with comparing gating obtained from live/dead reagents with this lymphocyte gate. The live/dead staining also confirms that lymphocytes treated with PBS or 25 ng/ml IL-15 concentrations have over 97% viable lymphocytes (**Figure 1 Supplementary section**). The purity of the NK, NKT, and CD8 T cells is shown in **Figure 2 Supplementary section**.

### IL-15 Is Superior to IL-2 in Increasing Cell Doubling Capacity in NK, NKT-Like, and CD8 T Cells

The effect of IL-15 and IL-2 on the cell doubling capacity was investigated using CFSE cell proliferation assay. **Figure 2** shows the effects of IL-2 and IL-15 on NK, NKT-like and CD8 T cell proliferation as expressed by the geometric mean fluorescence of CFSE after 7 days. Panel A displays the flow cytometric gating, panel B displays the normalized geometric mean fluorescence obtained with different concentrations of IL-2 and IL-15, and panel C shows the number of cell divisions observed above that of the control cell population (with no cytokines). The numbers of cell divisions were calculated from the geometric mean fluorescence intensity of the cells by using 1 cell division as equivalent to a 50% decrease in the geometric mean of CFSE. These results showed that as the concentration of IL-15 increased there was a significant decrease in this geometric mean fluorescence, with a maximal decrease at 25ng/ml IL-15 for NK cells (p=0.004) and at 50ng/ml IL-15 for NKT-like and CD8^+^ T cells (p=0.0001 and n=6; p=0.0026, respectively). These results correspond to a maximal four extra divisions in NK cells and seven divisions in NKT-like cells. In CD8 T cells there is a maximal extra three divisions with IL-15. For IL-2, significant decreases in geometric mean and the subsequent increased cell-division (maximal 2.49, 2.8, and 2.2 in NK, NKT-like and CD8 T cells, respectively) were only observed at 500–1000 ng/ml in each of the three cell types (n=6, p<0.001 by one way anova and post-hoc Newman Keuls).

### IL-15 Upregulates Telomerase TERT in NK, NKT-Like, and CD8 T Cells at Lower Doses Than IL-2

**Figure 3:**
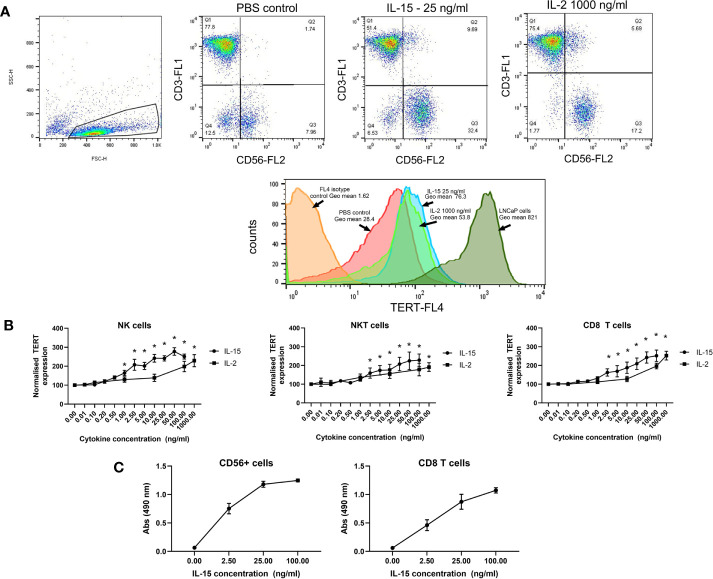
Effects of IL-15 and IL-2 on the expression of TERT in NK, NKT, and CD8 T cells in non-adherent PBMCs after 7 days culture. **(A)** shows dot plots and histograms displaying TERT as detected by flow cytometric analysis using Alexa-Fluor647 conjugated anti-TERT antibodies. Cells were also stained with fluorophore conjugated anti-CD8, anti-CD56, and anti-CD3 to distinguish the NK, NKT-like, and CD8 T cell populations. In **(B)**, results are expressed as the TERT geometric mean values from each experiment normalized to the PBS control. IL-15 induces significant increases in TERT in NK, NKT, and CD8 T cells starting at concentrations of 2.5 ng/ml. IL-2 also significantly increases TERT expression at concentrations from 100 ng/ml to 1000 ng/ml. Results represent means ± SEM from six separate experiments (six donors)(* p < 0.05 by one-way ANOVA and Newman Keuls post-hoc analysis). **(C)** shows telomerase activity in CD56^+^ and CD8+ T cells. measured *via* a TRAP assay after 7 days incubation with IL-15. Four concentrations of IL-15 were used: 0, 2.5, 25 and 100 ng/ml, with six repeats carried out. Results showed that as the concentration of IL-15 increased there was a rise in absorbance, indicating telomerase activity in the CD56+ and CD8 T cells.

Telomerase expression was measured by the detection of TERT in the effector cells. **Figure 3A** shows flow cytometric dot plots and histograms showing the expression of TERT on the 3 effector cell types, NK, NKT-like, and CD8 T cells. All three cell types express TERT without cytokine activation. An LNCaP cell line which has been shown previously to highly express HTERT (33) has been stained with HTERT as a positive control and is shown in the histogram for comparison of its HTERT expression to NK cells. The geometric mean of expression, signifying the intensity of expression per cell, is increased significantly in all three cell types by IL-15 compared with without any cytokines after 7 days (**Figure 3B**): In NK cells, expression is increased from 2 fold at 2.5 ng/ml up to 2.8 fold at 50 ng/ml (p<0.01). In NKT-like cells, expression is increased from a mean of 1.7 fold at 2.5 ng/ml up to 2.2 fold at 100 ng/ml (p<0.01). In CD8 T cells, TERT is increased from 1.6 fold at 2.5 ng/ml to 2.6 fold at 100 ng/ml (p<0.01). IL-2 treatment resulted in a more modest increase in TERT expression: In NK cells, expression increased significantly by 2 fold only at 100–1000 ng/ml and with CD8 T cells, increases were seen up to 100–1000 ng/ml by up to 2.5 fold (p<0.01). In NKT-like cells, IL-2 treatment produced similar increases to IL-15 (up to a 2.5 fold increase from untreated cells) but again, these were at higher doses (100–1000 ng/ml), (p<0.01).

To further confirm the efficacy of IL-15 in increasing activity of telomerase, we performed a TRAP assay on isolated CD56+ cells and CD8 T cells. **Figure 3C** shows that telomerase activity, as measured by the 490 nm absorbance of reactions from the telomerase PCR ELISA, is increased by IL-15 in both CD56+ cells and CD8 T cells.

### IL-15 and IL-2 Induce Differential Phosphorylation of Proteins From the Three Signaling Pathways JAK/STAT, P13/AKT, and RAS/MAPK

Cell lysates obtained from NK, NKT-like and CD8 T cells treated with IL-15 or IL-2 were used in a Human Phosphokinase array protein array to assess phosphorylation of a range of proteins in the three signaling pathways impacted by IL-2 and IL-15 (JAK/STAT, P13/AKT, and RAS/MAPK). From the array analysis, two membrane images were developed displaying two sets of dots that each correlated to a different protein.

**Figure 4:**
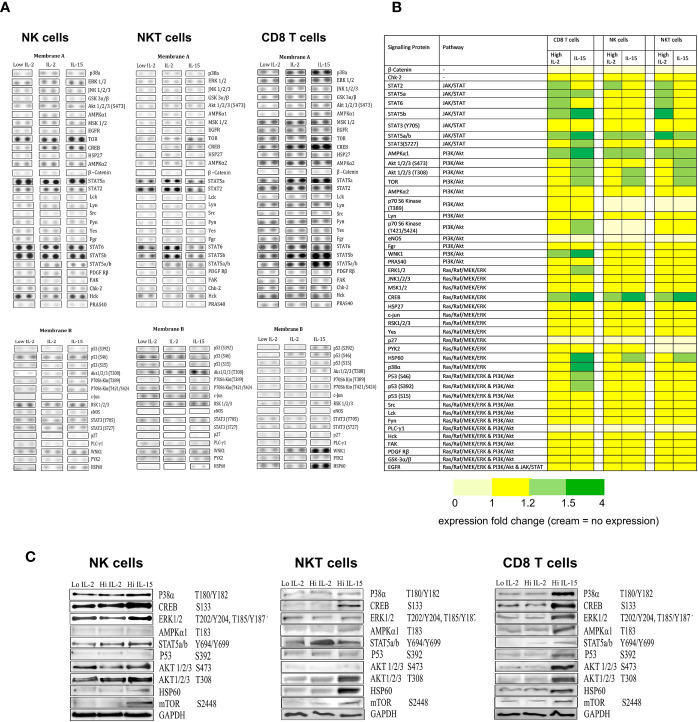
The effects of IL-15 and IL-2 on the expression of phosphorylation of proteins from the three signaling pathways JAK/STAT, P13/AKT, and RAS/MAPK. **(A)** shows membrane dot images developed from probing of the three cell types (NK, NKT, and CD8 T cells) treated with 1ng/ml IL-2 with the two membranes from a representative donor. The intensity of the dots relates to the detection of phosphorylated protein intensity within a certain sample. **(B)** displays a heatmap of the protein array collated from repeated experiments (n=4), with the proteins arranged by their signaling pathways and with colors indicating the degree of upregulation of phosphorylated proteins as calculated by densitometry carried out on the developed membranes. Yellow represents no change in expression compared to the low dose IL-2 control. Light green = protein expression increased by 1.1 fold -1.5 fold. Dark green = expression increased by more than 1.5 fold. Cream indicates no protein expression. **(C)** displays western blots of proteins selected from the array that showed increased phosphorylation (or upregulation of protein in the case of Hsp60).

From **Figures 4A, B**, NK cells show the least changes in phosphorylation of proteins from the array: IL-15 treatment increases Creb, Stat5a/b, Tor, AMPK α1, AKT (T308), and Hsp60 protein phosphorylation intensities above the low IL-2 control samples, whereas 1000 ng/ml IL-2 increases Creb, AMPK α1, Stat5a/b, and Stat2 phosphorylation. In NKT cells, IL-15 also increases Creb. Stat5a/b, Tor, AKT (T308) AMPKα1, and Hsp60 whereas IL-2 increases Stat2, AMPKα1, Stat5a, Stat5a/b, Stat5b, Stat6, and Creb. CD8 T cells display higher levels of phosphorylated proteins at the baseline (1ng/ml IL-2) level. IL-15 upregulates Creb, p38, ERK1/2, AKT (S473), AKT (T308), AMPKα1, p53 (s392), (s46), WNK1, Hsp60, Stat2, Stat5a, Stat5b, Stat5a/b, Stat6 in CD8 T cells, whereas IL-2 only upregulates AMPKα1, Creb, Stat2, Stat5a, Stat5b, Stat5a/b, and Stat6.

When we carried out western blots of selected proteins to confirm changes in the array (**Figure 4C**), IL-15 activity was confirmed for Creb, Stat5a/b, Tor, and Hsp60 in NK cells. In NKT cells, there was additional activity confirmed for AKT (T308) and in CD8 T cells, additional activity was seen with p53, ERK1,2,3 p38a and AMPKα1. For IL-2, only Stat5a/b activity was confirmed with the western blot, with no increases seen in Creb or AMPKα1.

### Effects of Inhibitors of the Three Signaling Pathways on TERT Expression in the Three Cell Types

**Figure 5:**
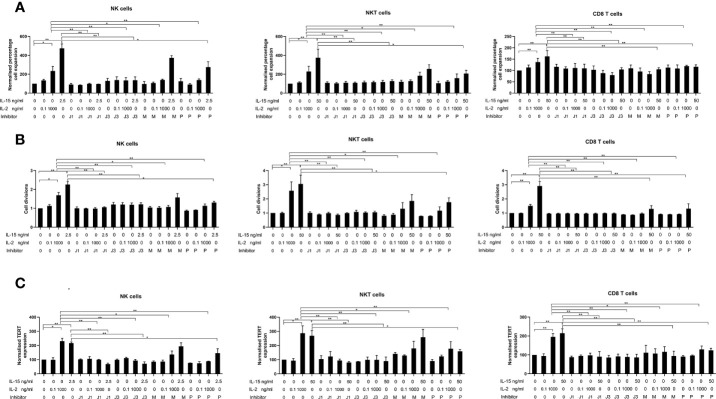
Effects of inhibitors of JAK1, JAK3, MEK/ERK, and PI3/AKT on the upregulation of expression of TERT **(A)**, cell expansion **(B)**, and cell divisions from CFSE data **(C)** in NK, NKT-like and CD8 T cells. Non-adherent PBMCs were aliquoted at 8 x 10^5^ cells/ml in 24-well plates together with the optimal concentrations of cytokines found to increase TERT expression for each cell type (2.5 ng/ml IL-15 and 1000 ng/ml IL-2 for NK cells, 25 ng/ml IL-15 and 1000 ng/ml IL-2 for NKT-like cells and 25 ng/ml IL-15 and 1000 ng/ml IL-2 for CD8 T cells), or controls (PBS)]. Cells were incubated for 7 days either with cytokines/control only or in the presence of inhibitors of each of the three signaling pathways known to be impacted by IL-2/IL-15 [CP690550 for JAK1 (J1) and JAK3 (J3) inhibition dependent on concentration, LY-294002 hydrochloride (P) for PI3K/AKT inhibition and U0126 (M) for inhibition of MEK/ERK]. Results are expressed as the normalized geometric mean expressions of TERT, normalized cell expansion, and normalized numbers of cell divisions and are the means +/- SEM from six separate experiments. The comparisons are between the each of the no inhibitor conditions (PBS, low IL-2, high IL-2 and IL-15) and the corresponding condition with each of the inhibitors. (*p < 0.05, **p < 0.01 by one-way ANOVA and Newman Keuls post-hoc analysis).

**Figure 5** displays bar charts of TERT expression in the three cell types where cells were treated with the inhibitors from the three signaling pathways JAK/STAT, P13/AKT and RAS/MAPK. The results are expressed as normalized geometric mean values of TERT, normalized cell expansion and normalized cell divisions obtained from the geometric mean values of the CFSE staining obtained from the flow cytometric analysis. Without inhibitors, TERT expression is increased by IL-2 and IL-15 by 2-2.5 fold in the three cell types as previously shown in the TERT experiments. All inhibitors of the three pathways (CP690550, LY-294002 hydrochloride, and U0126) abrogated IL-2 induced increases in TERT expression in the three cell types. The CP690550 inhibitor (used at two different doses to inhibit JAK1 and JAK3 signaling), also abrogated IL-15 induced increases in TERT expression in all three cell types. The MEK/ERK inhibitor significantly reversed IL-15 mediated increases in TERT expression only in CD8 T cells. The P13K/AKT inhibitor LY-294002 hydrochloride partially inhibited IL-15 increases in TERT expression in NK cells and abrogated its expression in NKT cells and CD8 T cells. Therefore all three pathways are involved in the IL-2 mediated increases in TERT in the three cell types, whereas IL-15 mediated increases in TERT involve all three pathways only in CD8 T cells: PI3K and JAK1/3 are involved in NKT cell TERT upregulation and in NK cells, only JAK1/3 inhibition fully inhibits increases in TERT expression with IL-15. Results obtained from the CFSE and expansion evaluations of the different cell types concur with the patterns of HTERT inhibition with the three inhibitors therefore showing that HTERT expression, cell expansion and division are similarly affected by the inhibition of the three pathways.

## Discussion

Effector NK cells, CD8 T cells and NKT-like cells have been shown to effectively kill tumor cells *in vivo* when adoptively transferred. Both the systemic levels of effector NK, NKT-like and CD8 T cells and their infiltration and activity in tumors correlates with patient survival in a number of cancer types (34–38). IL-2 and IL-15 have been identified as agents that can expand these cells in protocols where they are either injected systemically or used to treat effector cells that are then adoptively transferred. Identifying the optimal doses and understanding the mechanisms by which these two agents enhance effector cell proliferation is critical to their successful use in patients.

Our study shows that IL-15 is clearly superior to IL-2 in its expansion and cell doubling capacity of NK, NKT-like, and CD8 T cells. Doses of IL-15 required for expansion and cell doubling are between 50 and 100 times less than for IL-2 with significant expansion occurring from 0.5 ng/ml in NK cells, 1 ng/ml in NKT cells, and 25 ng/ml in CD8 T cells, whereas IL-2 only increases expansion from 100 ng/ml in NK and NKT cells and at 1000 ng/ml in CD8 T cells. These higher doses of IL-2 can also result in the production of Tregs (39). We have previously shown that Tregs are not upregulated by IL-15 at doses of 25 ng/ml (16). It is important to consider that optimal doses of IL-15 for proliferation of NK cells and NKT-like cells are far lower than for CD8 T cells, where a minimal 25 ng/ml is required for significant effects.

We wished to determine whether IL-15 upregulated telomerase activity and expression in NK and NKT-like cells in addition to CD8 T cells. Others have shown that the NK cell line, NK-92 cells expresses TERT upon stimulation with IL-2 (40) and that TERT transfection of NK cells can increase the longevity of the cells to at least 1 year when stimulated with K562 feeder cells expressing membrane-bound 4-1BB and IL-15 (41). In our study, when comparing TERT expression in CD8, NK, and NKT cells, we saw an upregulation of expression of the protein in all three cell types in PBMCs from normal human donors. This indicates that the replicative and lifespan potential impacted by TERT activity in each of these three cell types are similarly affected by IL-15. IL-15 upregulates TERT expression in NK, and NKT cells to a similar degree to that of CD8 T cells with a maximal 2.5 fold increase in the intensity of expression in the three cell types. The increases in TERT are concomitant with the increased cell doubling as observed with changes in CFSE intensity. IL-2 also upregulates TERT in the three cell types, but only at doses of 100–1000 ng/ml and the increase is less pronounced (a maximal 2-fold increase at 1000 ng/ml).

IL-15 upregulated HTERT in our experiments, to a maximum of 300% (3-fold). Although the expression of HTERT mRNA has been shown to increase by 8-fold in lymphocytes active by CD3/CD28 (42), and others have reported increases in protein of a similar magnitude using western blotting (40), westerns are much more sensitive than flow cytometry so they are not a direct comparison and an upregulation of mRNA may not translate to the same increase in protein.

The TRAP assay also confirms that the activity of telomerase is upregulated in both CD56+ and CD8 T cells to a similar degree with IL-15. IL-15 could therefore be used as an alternative to TERT transfection to increase the longevity of the three cell types.

Telomere length is also a parameter that is used to analyze telomerase activity. We considered carefully about including this data in our study and assessed different methods for obtaining the data for telomerase length. However, from our review of the methods and the results obtained from telomerase length analysis there is a large consensus that a) telomere length is a very variable parameter and is influenced by many environmental factors apart from the effects of cytokines, such as hormonal factors, age, sex of the individual donor (43) and b) the length of telomeres in individual leukocyte cells varies widely (44) so that the mean telomerase length may not change with our agents and the statistical power required to allow for the variance would mean testing many more donors than is practically possible. A previous study with lymphocytes examined telomere length with no changes recorded either for activation with IL-2 or for inhibition of activation with signaling inhibitors and this may be due to the above factors (40).

When we investigated the phosphorylation of proteins of the signaling pathways induced by IL-15 and IL-2, we found clear differences in the activation induced by IL-15 and IL-2 among the three cell types. In NK cells and NKT-like cells, IL-15 stimulated phosphorylation of a few selected proteins from the three pathways: Creb. Stat5a/b, Tor, AMPKα1, AKT (T308), and Hsp60. However, in CD8 T cells, there was increased phosphorylation of a larger number of proteins from the array: Creb, p38, ERK1/2, AKT (S473), AKT (T308), AMPKα1, p53 (s392), (s46), Tor, WNK1, Hsp60, Stat2, Stat5a, Stat5b, Stat5a/b, Stat6. Treatment with the high dose of IL-2 also changed phosphorylation of proteins in the three cell types, with significant changes in, AMPK α1, Creb, Stat2, Stat5a, Stat5b, Stat5a/b, and Stat6 in NKT-like and CD8 T cells and Creb, AMPK α1, Stat5a/b, and Stat2 in NK cells. IL-15’s effects on the proteins Creb, Stat5a/b, AKT, mTor, and Hsp60 were confirmed from the western blots of the three cell types and the effects on p38a, ERK and p53 were confirmed from western blots of the CD8 T cells. However, for IL-2, the results of the array were not confirmed for the western blots, with no change in Creb or AMPK α1 in the three cell lines.

The signaling array results confirm that IL-15 impacts signaling of a larger number of proteins in the three cell types than IL-2, which may explain its stronger activity, compared to IL-2 in the three cell types in cell expansion, TERT activity and cell division. The strong induction of Creb, MTOR and Hsp60 in all three cell types by IL-15 and not IL-2 may be a significant finding in the possible greater activity of IL-15 compared to IL-2 in NK, NKT, and CD8 T cell proliferation. The cAMP response element binding protein (CREB) is a well-known phosphorylation-activated transcription factor and its activation has been shown promote proliferation and survival and differentially regulate Th1, Th2, and Th17 responses in T and B cells (45). Furthermore it has been shown to inhibit the proliferation of T regulatory cells, which may be a possible contributor to the lack of Treg proliferation by IL-15 (46). Mammalian target of rapamycin (mTOR) has been previously shown to be important in the development and activation of NK cells by IL-15 (47) and also increases the migration of resting CD8 memory T cells to resident tissues (48). It is also known that mTOR regulates telomerase activity at the translational and post-translational level and that the inhibitor of mTOR, rapamycin, can inhibit telomerase activity (49).

The heat shock protein Hsp60 is expressed in eukaryotic cells as a response to a variety of stress stimuli, e.g. temperature increase, nutrient deprivation, exposure to toxic chemicals, inflammatory responses, and allograft rejection and it is known to bind to HLA-E to attenuate inhibition of NK responses through CD94/NKG2A recognition (50). The expression of Hsp60 may therefore increase the activity of NK cells through this mechanism. The heat shock proteins HSP90 and HSP70 have also been shown to be critical for NK cell function (51, 52) and it is known that Hsp90 forms a complex with TERT to increase the stability and activity of telomerase (53).

When we investigated the importance of the three signaling pathways JAK/STAT, PI3/AKT and RAS/MAPK in the increase in TERT expression mediated by IL-2 and IL-15, we showed that whereas all three pathways are involved in the increase through IL-2 in all three cell types, IL-15 mediated increases are dependent on JAK1 and JAK3 and PI3K/AKT in NK cells and NKT-like cells and all three pathways in CD8 T cells. The patterns of HTERT inhibition closely match the patterns of the inhibition of cell expansion and cell division. This finding is in line with additional proteins impacted in the Ras/Mek/pathways (ERk1/2 and p38a) in CD8 T cells by IL-15.

The dependence of TERT expression on all three signaling pathways with IL-2 and with IL-15 in CD8 T cells, suggests that any therapies that inhibit MEK/ERK pathways used in cancer patients are likely to affect proliferation of CD8 T cells to a greater extent than NK cells or NK-like cells.

Our study therefore demonstrates that IL-15 is preferable to IL-2 as a proliferative tool for preparing NK, NKT, or CD8 T cells for adoptive therapy and may be an alternative to the use of TERT transfection for increasing the replicative potential of these cells.

## Data Availability Statement

The original contributions presented in the study are included in the article/**Supplementary Material**. Further inquiries can be directed to the corresponding author.

## Author Contributions

CG obtained funding, created project and study, supervised staff and students, and designed experiments. PD obtained funding, recruited students to project, and developed concept. FW, SN, and AR carried out experiments and analyzed and interpreted data. CS and OE supervised FW and SN and interpreted and critiqued data. EP read and critiqued manuscript and interpreted data. All authors contributed to the article and approved the submitted version.

## Funding

The authors acknowledge financial support from Prostate Cancer Research (charity grant 6938) and Prostate cancer UK (grant PA-012).

## Conflict of Interest

The authors declare that the research was conducted in the absence of any commercial or financial relationships that could be construed as a potential conflict of interest.

## Supplementary Material

Supplementary Figure 1 Gating strategy for live cells and viability of lymphocytes with IL-15. To confirm that the gate we chose for lymphocytes for all flow cytometry experiments corresponded to live cells, we set up non-adherent lymphocytes for 7 days with either PBS or IL-15 at different concentrations. We then stained different lymphocyte populations with CD3, CD56 and CD8 T cells and included a live/dead stain APC-Cy7 stain (Thermofisher, UK). Panel **(A)** shows the whole lymphocyte population on forward and side scatter without gating and the corresponding channels showing the live/dead stain with fluorescence as APC-Cy7-A on the near Infrared channel on the FACs canto. In Panel **(B)**, when we gate the lymphocytes using our gate selection, this corresponds to the gating of the live cells with over 96% viable cells. Panel **(C)** shows that viability of the lymphocytes is not affected with IL-15 or IL-2.

Supplementary Figure 2 Dot plots showing the purity of **(A)** NKT, **(B)** NK and **(C)** CD8 T cells isolated from the Miltenyi kits. Cells were isolated by Miltenyi kits as described in the materials and methods and purity of the populations was assessed by flow cytometry. Purity was recorded above 95% in line with the standard kit isolation recommendations.
